# Hepatitis A Strain Linked to the European Outbreaks During Gay Events between 2016 and 2017, Identified in a Brazilian Homosexual Couple in 2017

**DOI:** 10.3390/v11030281

**Published:** 2019-03-20

**Authors:** Vinicius M. Mello, Barbara V. Lago, Paulo S. F. Sousa, Francisco C. A. Mello, Caroline B. Souza, Laura C. M. Pinto, Cleber F. Ginuino, Carlos A. S. Fernandes, Shirlei F. Aguiar, Lívia M. Villar, Elisabeth Lampe, Juliana G. Melgaço, Lia L. Lewis-Ximenez

**Affiliations:** 1 Viral Hepatitis Laboratory, Oswaldo Cruz Institute, Oswaldo Cruz Foundation, Rio de Janeiro RJ 21040-900, Brazil; paulosfsousa@gmail.com (P.S.F.S.); fcamello@gmail.com (F.C.A.M.); carolinebaldin@uol.com.br (C.B.S.); lauracmpinto@gmail.com (L.C.M.P.); cleber@ioc.fiocruz.br (C.F.G.); liviafiocruz@gmail.com (L.M.V.); elisabeth.fiocruz@gmail.com (E.L.); juliana.melgaco@gmail.com (J.G.M.); lialewis.fiocruz@gmail.com (L.L.L.-X.); 2 Institute of Technology in Immunobiologics–Bio-Manguinhos, Oswaldo Cruz Foundation, Rio de Janeiro RJ 21040-900, Brazil; 3 Hepatitis Division, Central Public Health Laboratory Noel Nutels, Rio de Janeiro RJ 20231-092, Brazil; cas.fernandes@gmail.com (C.A.S.F.); shirleideaguiar@gmail.com (S.F.A.)

**Keywords:** Hepatitis A virus (HAV), European strain, HAV in Brazilian MSM

## Abstract

Hepatitis A virus (HAV) outbreaks among men who have sex with men (MSM) have been reported worldwide and associated primarily with sexual transmission through oral-anal sex. Here, we provide the molecular and evolutionary description of a European strain, linked to HAV outbreaks among MSM, detected in a Brazilian homosexual couple. Bayesian analysis provided evidence that the viral isolates were introduced in Brazil from Spain between the end of 2016 and the beginning of 2017.

## 1. Introduction

Hepatitis A virus (HAV) infection, transmitted by the fecal-oral route, occurs mainly through person-to-person contact or ingestion of contaminated water or food [1]. Household members and close contact with children were identified, for many years, as the most common risk factor, being the latter responsible for several HAV outbreaks. However, in the early 1980s sexual transmission of HAV was reported [2], showing an increase in incidence over time [3] among men who have sex with men (MSM) and accounting for isolated outbreaks in different countries [4,5]. Risk factors associated with HAV infection among MSM were oral-anal intercourse and group sex [5,6]. As of 2016, an increase in incidence of HAV infection among MSM was observed in many countries, with many outbreaks occurring following social events such as gay festivals, where distinct strains of genotype IA, as VRD_521_2016 and RIVM-HAV16–090 were identified in more than 3000 cases in European Union [7,8,9,10,11,12]. In Brazil, an increase of 128% in the incidence of HAV infection among males, 20–39 years/old, reporting oral-anal sex was described by the Ministry of Health in their 2017 annual Epidemiological Bulletin [13]. Despite these considerations, there are scarce studies in Brazil on HAV transmission among MSM. Our findings identified for the first time the European strain VRD_521_2016 in a homosexual couple, possibly introduced in Brazil at the end of 2016. 

## 2. Materials and Methods

### Investigation

A homosexual male couple received medical assistance at the Viral Hepatitis Ambulatory/Oswaldo Cruz Foundation, Rio de Janeiro in September 2017. They were assessed for major risk factors, socio-behavioral practices, and for the presence of sexually transmitted infections (STIs). Samples were collected from and were tested for hepatitis A, B (HBV), C (HCV), and E (HEV) serological markers as follows: HAV IgM and IgG antibodies, HBsAg and HBeAg antigen; HBc IgM, IgG, HBe and HBs antibodies, HCV antibodies (LIAISON^®^ XL, DiaSorin, Italy) and HEV antibodies (recomWell HEV IgG/IgM, Mikrogen, Germany). They were also tested for HIV (LIAISON^®^ MUREX HIV Ab/Ag, DiaSorin, Italy and Imunoblot Rápido DPP^®^ HIV 1/2, Bio Manguinhos, Brazil) and syphilis (Immuno-Rápido Sífilis^®^, Wama, Brazil). HAV RNA was detected through a qualitative reverse transcriptase PCR. Phylogenetic and Bayesian evolutionary analyzes were conducted with a dataset composed of 85 VP1-2A region sequences with 295 nucleotides from genotype IA, representing the main HAV worldwide outbreaks, with known sample collection date retrieved from Genbank. Additionally, six Brazilian samples from 2015 were sequenced to genomic analyzes. Calculations of the time of the most recent common ancestor (tMRCA) of internal nodes were estimated under an uncorrelated lognormal relaxed molecular clock model. Markov Chain Monte Carlo (MCMC) was run for 100 × 106 generations using General Time Reversible model with gamma distributed rate heterogeneity (GTR+G) (BEAST software; version 1.8.10; http://beast.community/). The convergence of the MCMC (estimated sum of squares >200) was assessed using Tracer version 1.7 (http://beast.community/tracer). Uncertainties in the parameters were assessed by 95% Highest Posterior Density (HPD) interval. The consensus tree was estimated by the TreeAnnotator program v1.6.1.

All subjects gave their informed consent for inclusion before they participated in the study and the protocol was approved by the FIOCRUZ Ethics Committee with number: 50230015.0.0000.5248. 

## 3. Results

**Figure 1:**
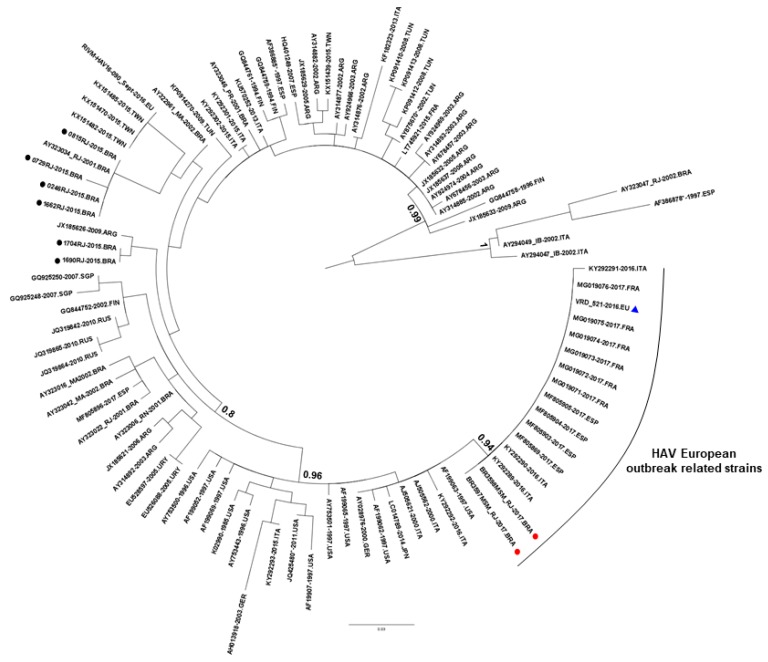
Phylogenetic tree performed by using the Maximum Likelihood method, with 100 HAV genotype IA isolates. The European strain VRD_521_2016 is marked with (▲). The study sequences related to the European outbreak (GenBank accession numbers: MK170458 and MK170459) are marked with a red dot (●), Brazilian strains from 2015 (GenBank accession numbers: MK170460 to MK170463, MK170465, and MK170466) are represented with black dots (●), and the most relevant approximate.

The couple, 36 and 37 years old, reported clinical symptoms such as: jaundice, whitish stool, dark urine, abdominal pain, nausea, vomiting, fever, and weight loss. They reported a three-year open relationship, unprotected anal sex, oral sex, oral-anal, and group sex with two/three different partners. Both denied exposure to other possible HAV sources, such as recreational swimming, shellfish ingestion, drinking unfiltered water in the last month, as well as having any contact with children or individuals with known hepatitis. The couple also denied having travelled abroad. The patients were negative for HCV and HEV tests. One of them presented serological markers of previous HBV infection (anti-HBc+/anti-HBs+). The patients reported previous syphilis and were positive for HIV, confirmed by chemiluminescence immunoassay and immunoblot. Both were positive for HAV IgM antibodies and HAV RNA. Phylogenetic analysis demonstrated strong association (100% sequence homology) between the couple’s samples and formed a monophyletic group with genotype IA strains identified in European outbreaks among MSM (>99.7% sequence homology). Although genotype IA is the most prevalent in Brazil, the study samples were not genetically related to other Brazilian strains (genetic distance: 0.013) (Figure 1).

**Figure 2:**
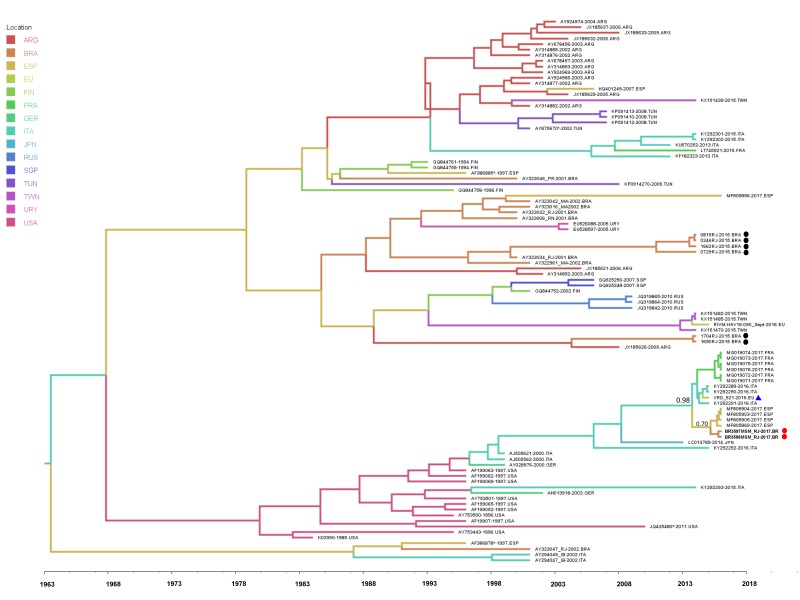
Time-scaled Bayesian MCMC tree of the hepatitis A virus from different countries between the years 1994 and 2017. Branches are colored according to the most probable location of their descendent nodes. The European strain VRD_521_2016 is marked with (▲). The study sequences related to the European outbreak are marked with a red dot (●), Brazilian strains from 2015 are represented with black dots (●). Color code is indicated in the upper left legend. ARG: Argentina; BRA: Brazil; ESP: Spain; EU: Europe Hepatitis A strain; FIN: Finland; FRA: France; GER: Germany; ITA: Italy; JPN: Japan; RUS: Russia; SGP: Singapore; TUN: Tunisia; TWN: Taiwan; URY: Uruguay; USA: United States.

The mean nucleotide substitution rate was estimated in 1.56 × 10^−3^ substitutions/site/year (95% HPD, 6.76 × 10^−4^ to 2.61 × 10^−3^) under the best fit model. Despite the short time since the beginning of the European HAV outbreaks among MSM, we calculated the time the strain VRD_521_2016 was introduced in Brazil and its possible dispersal pattern. According to the Bayesian analysis (pp: 0.98), the most plausible route of the VRD_521_2016 to Brazil was throught Europe. Despite the uncertainty about the country of origin of the MSM Brazilian sequences (pp: 0.7), our analysis suggests that viral isolates might have been introduced in Brazil from Spain between the end of 2016 and the beginning of 2017. The root of the VRD_521_2016 clade dated back to *2013* (Figure 2).

## 4. Discussion

Due to the recent increase in HAV infection in Brazil, the Ministry of Health has extended the indication of HAV vaccine to individuals who practice oral-anal sex, especially among MSM [14]. However, the strains responsible for these cases have not yet been characterized.

The couple enrolled in this study reported unprotected anal-oral sex and multiple sexual partners. Studies in HAV outbreaks among MSM have shown such practices to be common among the infected cases [7,9], reinforcing the sexual practice as the probable transmission route. In addition, coinfection with other STIs, such as HIV, syphilis and hepatitis B have been reported suggesting a high incidence of unprotected sex thus increasing exposure and promoting further transmission to enteric viruses such as HAV [8,10,11]. The couple studied here presented concurrent HAV and HIV antibodies and serological evidence of past syphilis infection. Studies have shown that HIV-infected patients have elevated risk for HAV co-infection [15,16]. Likewise, HIV co-infection can exacerbate HAV-associated liver damage and extend the HAV fecal excretion period [16,17], increasing the risk of spread to others [12], through water sources in areas that lack adequate sanitation. 

Phylogenetic analysis showed a monophyletic cluster of genotype IA (aLRT = 0.94) enrolling the couple’s samples and sequences from the European MSM outbreaks, demonstrating that this strain (VRD_521_2016) is circulating in Rio de Janeiro, Brazil, and may or may not be limited to MSM. The mean evolutionary rate, estimated in 1.56 × 10^−3^ substitutions/site/year (95% HPD, 6.76 × 10^−4^ to 2.61 × 10^−3^), is consistent with other studies on HAV genotype IA VP1-2A (1.21 × 10^−3^ substitutions/site/year) and HAV genotype I VP3-VP1-2A (5.56 × 10^−4^ substitutions/site/year) [18,19]. The Bayesian inference revealed that the most recent common ancestor of the strain VRD_521_2016 might have been originated around 2013 in Europe (root of the clade) and may be introduced in Brazil between the second half of 2016 and the beginning of 2017. This strain was identified in July 2016 among UK MSM who travel to Spain [7,20], and then detected in several MSM outbreaks in European countries [7]. Approximately, 500,000 tourists visited Brazil due to the Olympic and Paralympic Games that took place between August to October 2016 in Rio de Janeiro, [21] leading to an intense flow of people from several continents. Thus, it is possible that the strain VRD_521_2016 was introduced in Brazil during the Olympics and spread rapidly through sexual practices among MSM, being responsible for the increase in HAV infection in adult men in 2017 [13]. Studies have shown that demographic changes are often associated with the introduction of new pathogens, as demonstrated by the spread of the ZIKA virus in 2015 [22]. Nevertheless, while this strain may have been implicated in the increase in HAV among men in 2017 in Brazil, further studies involving sequencing of a larger samples should be implemented to test and to reinforce this hypothesis.

This study reports the introduction of the HAV strain VRD_521_2016 in Brazil, possibly linked to the intense tourism promoted by the Olympic Games. This reinforces the urgent need for health authorities to improve access to HAV vaccine to high-risk adult groups. Moreover, education measures are useful to prevent the spread of HAV to key populations.
